# Supplementary material for: HIVprotI: an integrated web based platform for prediction and design of HIV proteins inhibitors
Source: J Cheminform. 2018 Mar 9;10:12. doi: 10.1186/s13321-018-0266-y (PMC5845081; doi:10.1186/s13321-018-0266-y)
Supplement: Supplementary file 2 — Additional file 2. Source code of HIVProtI web server. [file 13321_2018_266_MOESM2_ESM.zip › HIVprotI_Source-code/hivprotI/weka-3-6-6/doc/weka/classifiers/mi/MILR.html]

MILR


---


|  |  |  |
| --- | --- | --- |
| **Overview** \| **Package** \| **Class** \| **Tree** \| **Deprecated** \| **Index** \| **Help** \| **Weka's home** | |  |
| **PREV CLASS**   **NEXT CLASS** | **FRAMES**    **NO FRAMES**     **All Classes** |  |
| SUMMARY: NESTED \| FIELD \| CONSTR \| METHOD | DETAIL: FIELD \| CONSTR \| METHOD |  |


---


## weka.classifiers.mi Class MILR

```
java.lang.Object
  weka.classifiers.Classifier
      weka.classifiers.mi.MILR
```

**All Implemented Interfaces:**: java.io.Serializable, java.lang.Cloneable, CapabilitiesHandler, MultiInstanceCapabilitiesHandler, OptionHandler, RevisionHandler

---

``` public class MILR extends Classifier implements OptionHandler, MultiInstanceCapabilitiesHandler ```

Uses either standard or collective multi-instance assumption, but within linear regression. For the collective assumption, it offers arithmetic or geometric mean for the posteriors.


Valid options are:

```
 -D
  Turn on debugging output.
```

```
 -R <ridge>
  Set the ridge in the log-likelihood.
```

```
 -A [0|1|2]
  Defines the type of algorithm:
   0. standard MI assumption
   1. collective MI assumption, arithmetic mean for posteriors
   2. collective MI assumption, geometric mean for posteriors
```

**Version:**
:   $Revision: 5527 $

**Author:**
:   Eibe Frank (eibe@cs.waikato.ac.nz), Xin Xu (xx5@cs.waikato.ac.nz)

**See Also:**: Serialized Form

---

| **Field Summary** | |
| --- | --- |
| `static int` | `ALGORITHMTYPE_ARITHMETIC`             collective MI assumption, arithmetic mean for posteriors |
| `static int` | `ALGORITHMTYPE_DEFAULT`             standard MI assumption |
| `static int` | `ALGORITHMTYPE_GEOMETRIC`             collective MI assumption, geometric mean for posteriors |
| `static Tag[]` | `TAGS_ALGORITHMTYPE`             the types of algorithms |


**Constructor Summary**

`MILR()`


| **Method Summary** | |
| --- | --- |
| `java.lang.String` | `algorithmTypeTipText()`             Returns the tip text for this property |
| `void` | `buildClassifier(Instances train)`             Builds the classifier |
| `double[]` | `distributionForInstance(Instance exmp)`             Computes the distribution for a given exemplar |
| `SelectedTag` | `getAlgorithmType()`             Gets the type of algorithm. |
| `Capabilities` | `getCapabilities()`             Returns default capabilities of the classifier. |
| `Capabilities` | `getMultiInstanceCapabilities()`             Returns the capabilities of this multi-instance classifier for the relational data. |
| `java.lang.String[]` | `getOptions()`             Gets the current settings of the classifier. |
| `java.lang.String` | `getRevision()`             Returns the revision string. |
| `double` | `getRidge()`             Gets the ridge in the log-likelihood. |
| `java.lang.String` | `globalInfo()`             Returns the tip text for this property |
| `java.util.Enumeration` | `listOptions()`             Returns an enumeration describing the available options |
| `static void` | `main(java.lang.String[] argv)`             Main method for testing this class. |
| `java.lang.String` | `ridgeTipText()`             Returns the tip text for this property |
| `void` | `setAlgorithmType(SelectedTag newType)`             Sets the algorithm type. |
| `void` | `setOptions(java.lang.String[] options)`             Parses a given list of options. |
| `void` | `setRidge(double ridge)`             Sets the ridge in the log-likelihood. |
| `java.lang.String` | `toString()`             Gets a string describing the classifier. |

**Methods inherited from class weka.classifiers.Classifier**

`classifyInstance, debugTipText, forName, getDebug, makeCopies, makeCopy, setDebug`

**Methods inherited from class java.lang.Object**

`equals, getClass, hashCode, notify, notifyAll, wait, wait, wait`

**Field Detail**

### ALGORITHMTYPE\_DEFAULT

```
public static final int ALGORITHMTYPE_DEFAULT
```

:   standard MI assumption

    **See Also:**: Constant Field Values

---


### ALGORITHMTYPE\_ARITHMETIC

```
public static final int ALGORITHMTYPE_ARITHMETIC
```

:   collective MI assumption, arithmetic mean for posteriors

    **See Also:**: Constant Field Values

---


### ALGORITHMTYPE\_GEOMETRIC

```
public static final int ALGORITHMTYPE_GEOMETRIC
```

:   collective MI assumption, geometric mean for posteriors

    **See Also:**: Constant Field Values

---


### TAGS\_ALGORITHMTYPE

```
public static final Tag[] TAGS_ALGORITHMTYPE
```

:   the types of algorithms


**Constructor Detail**

### MILR

```
public MILR()
```


**Method Detail**

### globalInfo

```
public java.lang.String globalInfo()
```

:   Returns the tip text for this property

    :   **Returns:**: tip text for this property suitable for displaying in the explorer/experimenter gui

---


### listOptions

```
public java.util.Enumeration listOptions()
```

:   Returns an enumeration describing the available options

    :   **Specified by:**: `listOptions` in interface `OptionHandler` **Overrides:**: `listOptions` in class `Classifier`
    :   **Returns:**: an enumeration of all the available options

---


### setOptions

```
public void setOptions(java.lang.String[] options)
                throws java.lang.Exception
```

:   Parses a given list of options.

    :   **Specified by:**: `setOptions` in interface `OptionHandler` **Overrides:**: `setOptions` in class `Classifier`
    :   **Parameters:**: `options` - the list of options as an array of strings **Throws:**: `java.lang.Exception` - if an option is not supported

---


### getOptions

```
public java.lang.String[] getOptions()
```

:   Gets the current settings of the classifier.

    :   **Specified by:**: `getOptions` in interface `OptionHandler` **Overrides:**: `getOptions` in class `Classifier`
    :   **Returns:**: an array of strings suitable for passing to setOptions

---


### ridgeTipText

```
public java.lang.String ridgeTipText()
```

:   Returns the tip text for this property

    :   **Returns:**: tip text for this property suitable for displaying in the explorer/experimenter gui

---


### setRidge

```
public void setRidge(double ridge)
```

:   Sets the ridge in the log-likelihood.

    :   **Parameters:**: `ridge` - the ridge

---


### getRidge

```
public double getRidge()
```

:   Gets the ridge in the log-likelihood.

    :   **Returns:**: the ridge

---


### algorithmTypeTipText

```
public java.lang.String algorithmTypeTipText()
```

:   Returns the tip text for this property

    :   **Returns:**: tip text for this property suitable for displaying in the explorer/experimenter gui

---


### getAlgorithmType

```
public SelectedTag getAlgorithmType()
```

:   Gets the type of algorithm.

    :   **Returns:**: the algorithm type

---


### setAlgorithmType

```
public void setAlgorithmType(SelectedTag newType)
```

:   Sets the algorithm type.

    :   **Parameters:**: `newType` - the new algorithm type

---


### getCapabilities

```
public Capabilities getCapabilities()
```

:   Returns default capabilities of the classifier.

    :   **Specified by:**: `getCapabilities` in interface `CapabilitiesHandler` **Overrides:**: `getCapabilities` in class `Classifier`
    :   **Returns:**: the capabilities of this classifier **See Also:**: `Capabilities`

---


### getMultiInstanceCapabilities

```
public Capabilities getMultiInstanceCapabilities()
```

:   Returns the capabilities of this multi-instance classifier for the
    relational data.

    :   **Specified by:**: `getMultiInstanceCapabilities` in interface `MultiInstanceCapabilitiesHandler`
    :   **Returns:**: the capabilities of this object **See Also:**: `Capabilities`

---


### buildClassifier

```
public void buildClassifier(Instances train)
                     throws java.lang.Exception
```

:   Builds the classifier

    :   **Specified by:**: `buildClassifier` in class `Classifier`
    :   **Parameters:**: `train` - the training data to be used for generating the boosted classifier. **Throws:**: `java.lang.Exception` - if the classifier could not be built successfully

---


### distributionForInstance

```
public double[] distributionForInstance(Instance exmp)
                                 throws java.lang.Exception
```

:   Computes the distribution for a given exemplar

    :   **Overrides:**: `distributionForInstance` in class `Classifier`
    :   **Parameters:**: `exmp` - the exemplar for which distribution is computed **Returns:**: the distribution **Throws:**: `java.lang.Exception` - if the distribution can't be computed successfully

---


### toString

```
public java.lang.String toString()
```

:   Gets a string describing the classifier.

    :   **Overrides:**: `toString` in class `java.lang.Object`
    :   **Returns:**: a string describing the classifer built.

---


### getRevision

```
public java.lang.String getRevision()
```

:   Returns the revision string.

    :   **Specified by:**: `getRevision` in interface `RevisionHandler` **Overrides:**: `getRevision` in class `Classifier`
    :   **Returns:**: the revision

---


### main

```
public static void main(java.lang.String[] argv)
```

:   Main method for testing this class.

    :   **Parameters:**: `argv` - should contain the command line arguments to the scheme (see Evaluation)


---


|  |  |  |
| --- | --- | --- |
| **Overview** \| **Package** \| **Class** \| **Tree** \| **Deprecated** \| **Index** \| **Help** \| **Weka's home** | |  |
| **PREV CLASS**   **NEXT CLASS** | **FRAMES**    **NO FRAMES**     **All Classes** |  |
| SUMMARY: NESTED \| FIELD \| CONSTR \| METHOD | DETAIL: FIELD \| CONSTR \| METHOD |  |


---
